# Lateral interactions between CD276 and CD147 are essential for stemness in breast cancer: a novel insight from proximal proteome analysis

**DOI:** 10.1038/s41598-023-41416-7

**Published:** 2023-08-30

**Authors:** Yu Ri Seo, Junghyeon Lee, Han Suk Ryu, EunHee G. Kim, So Hyun Kim, Jieun Jeong, Hyeryeon Jung, YeoJin Jung, Han Byeol Kim, Yeon Hui Jo, Yeong Dong Kim, Min-Sun Jin, Yong Yook Lee, Kristine M. Kim, Eugene C. Yi

**Affiliations:** 1 Department of Molecular Medicine and Biopharmaceutical Sciences, Graduate School of Convergence Science and Technology and College of Medicine or College of Pharmacy, Seoul National University, Seoul, Republic of Korea; 2 Department of Bio-Health Convergence, Kangwon National University, Chuncheon, Republic of Korea; 3 Department of Systems Immunology, Division of Biomedical Convergence, College of Biomedical Science, Kangwon National University, Chuncheon, Republic of Korea; 4 Department of Pathology, Seoul National University Hospital, College of Medicine, Seoul National University, Seoul, Republic of Korea; 5 Department of Pathology, Bucheon St. Mary’s Hospital, College of Medicine, The Catholic University of Korea, Bucheon, Republic of Korea

**Keywords:** Proteomics, Mass spectrometry

## Abstract

Oncogenic cell-surface membrane proteins contribute to the phenotypic and functional characteristics of cancer stem cells (CSCs). We employed a proximity-labeling proteomic approach to quantitatively analyze the cell-surface membrane proteins in close proximity to CD147 in CSCs. Furthermore, we compared CSCs to non-CSCs to identify CSC-specific cell-surface membrane proteins that are closely interact with CD147 and revealed that lateral interaction between CD147 and CD276 concealed within the lipid raft microdomain in CSCs, confers resistance to docetaxel, a commonly used chemotherapy agent for various cancer types, including metastatic breast cancer. Moreover, we investigated the clinical relevance of CD147 and CD276 co-expression in HER2+ breast cancer (BC) and triple-negative breast cancer patients who underwent chemotherapy. We observed poor disease-free survival and Overall survival rates in patients of CD147 and CD276 (*p* = 0.04 and 0.08, respectively). Subsequent immunohistochemical analysis in independent cohorts of HER2+ BC support for the association between co-expression of CD147 and CD276 and a poor response to chemotherapy. Collectively, our study suggests that the lateral interaction between CD147 and its proximal partners, such as CD276, may serve as a poor prognostic factor in BC and a predictive marker for the critical phenotypic determinant of BC stemness.

## Introduction

Plasma membrane proteins play a crucial role in mediating responses to endogenous and environmental signals, thereby regulating diverse intracellular signaling events^1^. Recent evidence has highlighted the significance of lateral interactions among oncogenic membrane proteins that assemble within spatially restricted intracellular compartments, contributing to the development of aggressive phenotypes in cancer cells^2^. Although biochemical methods provide partial characterization of these oncogenic protein complexes, mapping the assembled components of these complex clusters in live cells presents experimental challenges that are difficult to overcome. The emergence of the in-situ enzyme-catalyzed proximal protein labeling approach has revolutionized the mapping protein complexes sequestered within intracellular compartments^3^. This technique enables the identification and characterization of cell-surface proteins assembling into well-defined domains, offering valuable insights into the organizational details of the dynamic cell-surface proteins at the molecular level. However, there has been limited investigation into the dynamic assembly of oncogenic cell-surface proteins at larger spatial scales associated with cancer cells, warranting further exploration and research efforts.

CD147, also known as EMMPRIN/basigin, is a cell-surface glycoprotein widely expressed in various cancer cell types^4,5^. Its upregulated expression plays a significant role in BC by contributing to cancer cell proliferation, metastasis, invasion, and chemoresistance^6–8^. This multifunctional transmembrane protein interacts with several oncogenic proteins, collectively contributing to cancer malignancy and enhance resistance to anticancer drugs^6^. Additionally, CD147 acts as a functional interacting partner for plasma membrane proteins such as epidermal growth factor receptor (EGFR), CD133, and CD44. It is proposed to induce the assembly of signaling complexes in lipid raft-like microdomains (LRMs), promoting survival or tumor initiation^9,10^. Moreover, CD147 is closely associated with lactate transporters, including monocarboxylic acid transporter 1 (MCT1) and MCT4, and plays a crucial role in their cell-surface expression and activities in solid tumors^11^. CD147 is an essential regulatory molecule in tumor progression and the development of chemotherapy resistance. However, the molecular events underlying CD147-induced oncogenic assembly and its association with BC stemness in cancer stem cells (CSCs) of BC remain unclear. Mapping the interaction networks of proteins that interact with CD147 within the membrane would provide insights into the organization of the CD147-interacting proteome as functional units. This information is crucial for comprehending the complex biological processes that lead to the cancer stemness phenotype.

In this study, we employed the proximal protein labeling method to identify cell-surface proteins that co-localized with CD147 in CSCs. These CSCs were generated from MDA-MB453 breast cancer cell lines transfected with Oct3/4 stem cell specific promoter. Previous studies by Sajithlal et al.^12^ have shown that the overexpression of Oct3/4 leads to an enriched cell population exhibiting the CD44^high^/CD24^low^ phenotype, along with chemoresistance and the ability to survive in acidotic environments.

Furthermore, Kang et al.^7^ conducted a quantitative surface proteome analysis of CSC-like cells and identified several unique overexpressed surface proteins, including CD147 and CD276. Building upon these findings, we performed a proximal proteome analysis using horseradish peroxidase (HRP)-conjugated anti-CD147 antibody (anti-CD147-HRP Ab). This analysis allowed us to identify 43 proteins in close proximity to CD147 in CSCs, including CD44, CD133, EGFR, MCT4, and CD276.

Importantly, we observed the formation of a cell-surface assembly in CSCs, wherein selected proteins such as CD44, CD133, EGFR, MCT4, and CD276 co-localized with CD147 in the LRMs. This lateral interaction was not observed in non-CSCs (NCSCs), highlighting its specific association with the CSC phenotype. Notably, while CD44 and CD133 are recognized as the breast CSC markers, CD276 has been reported to play a role in promoting tumorigenesis and drug resistance in various carcinoma cell lines^13–16^. Furthermore, CD147 is known to regulate BC invasion through association with CD44, EGFR, or P-glycoprotein^10,17^. In our study, we utilized target gene knockout (KO) systems and disrupted LRMs to further elucidate the role of the cell-surface assembly formed by CD147 and CD276 within LRMs is associated with BC stemness.

Additionally, we investigated the expression of CD147 and CD276 in BC tissues and found that their co-expression was significantly correlated with an unfavorable clinical outcome in patients with HER2+ BC and TNBC who were undergoing chemotherapy. These results highlight the clinical relevance of the CD147 and CD276 interaction and its impact on treatment response and disease progression.

Taken together, our data provide compelling evidence for the lateral interaction between CD147 and CD276, sequestered within LRMs, as a crucial determinant of the drug resistance phenotype in cancer stem cells (CSCs) and the pathological features of human BC. These findings contribute to our understanding of the molecular mechanisms underlying BC progression and offer potential targets for therapeutic interventions.

## Results

### Identification of CD147 proximal cell-surface proteins expressed in CSCs

**Figure 1:**
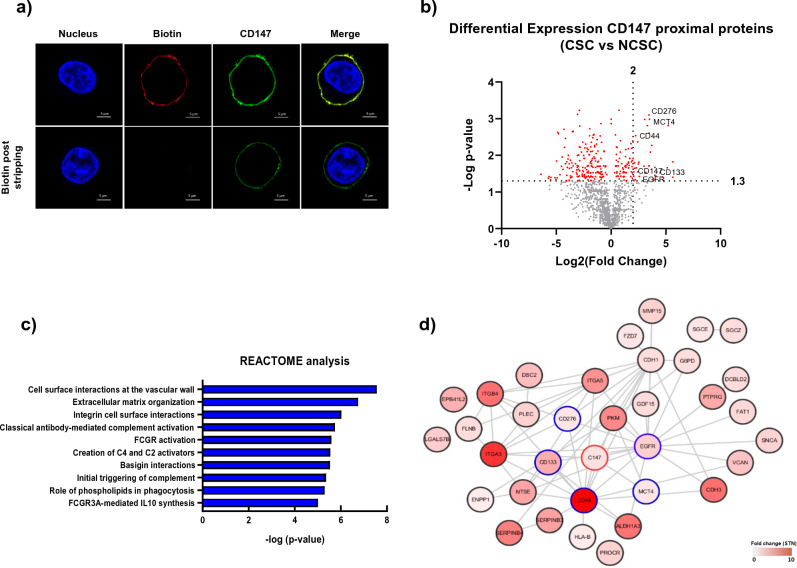
CD147 proximal proteins and their functional characteristics. (**a**) Confocal immunofluorescence images display the cell-surface biotinylation of CSCs using the anti-CD147-HRP Ab probe. Live CSCs were biotinylated with thiol-cleavable tyramide-biotin and H_2_O_2_, as described in the Method section. The cells were fixed and stained with Alexa647-conjugated F(ab’)2 goat anti-huIgG to visualize CD147 (green) and streptavidin-Texas Red-X conjugate to visualize biotinylated proteins (red). Biotin labels deposited on the cell surface were subsequently stripped by TCEP treatment, and stained cells are shown in the lower panel. (**b**) The volcano plot depicts the differential expression of genes encoding the CD147 proximal proteins in CSCs compared to NCSCs. Biotinylated proteins from both cell types, labeled with thiol-cleavable tyramide-biotin, were enriched through purification and fractionated by SDS-PAGE. In-gel tryptic digestion and LC–MS/MS analysis were performed, followed by label-free quantitation to identify CSC-specific CD147 proximal proteins. The volcano plot demonstrates the fold changes in abundance (on the x-axis) of CD147 proximal proteins in CSCs relative to the NCSCs. A total of 42 CSC-specific CD147 proximal proteins exhibiting statistically significant changes in abundance (log_2_ fold change ≥ 2.0; *p* < 1.3). (**c**) REACTOME analysis reveals the functional enrichment of CSC-specific CD147 proximal proteins. (**d**) The CD147 protein–protein interaction map is generated from the publicly available protein interaction data through the Search Tool in the Retrieval of Interacting Genes database.

To identify the surface proteins assembled in the vicinity of CD147 in CSCs, we employed the proximal proteome labeling approach^18^. We utilized either anti-CD147-HRP Ab or HRP-conjugated immunoglobulin G antibody (anti-IgG-HRP Ab) as a negative control, along with the tyramide-biotin reagent. To confirm the biotinylation of cell-surface proteins by the anti-CD147-HRP antibody probe, we performed immunofluorescence-imaging analysis of CSCs. The overlapping immunofluorescence images of CD147 and biotin deposition on the cell surface of CSCs indicated a prevalence of biotinylation on the cell surface of CSCs (Fig. 1a). Moreover, the intensity of the stained images also correlated with higher CD147 expression levels in CSCs. By stripping the biotins from the cell-surface-bound biotinylated molecules on CSCs, we further confirmed that the overlapping immunofluorescence image resulted from biotin deposition from the anti-CD147-HRP Ab probe (Fig. 1a).

To identify proteins uniquely associated with CD147 expressed in CSCs, we incubated an equal number of CSC and NCSC cells with either anti-CD147-HRP or the negative control anti-IgG-HRP Ab. After biotin deposition onto the cell surface, we isolated biotinylated proteins through streptavidin purification. Equal amounts of proteins (100 µg each) from CSCs, NCSCs, and the negative control were fractionated by SDS-PAGE. Subsequently, the proteins underwent in-gel tryptic digestion (see Supplementary Fig. S1) and were subjected to duplicate LC–MS/MS analyses. Peaks Studio ver. 8.5 protein database search identified 2,188, 2,667, and 1,875 proteins (false discovery rate ≤ 1%) in CSCs, NCSCs, and the negative control, respectively. Using spectral counting-based label-free quantification, we identified a total of 43 enriched and unique proteins (STN > 2.5, *p* < 0.01) in CSCs with at least two unique peptides and a false discovery rate of ≤ 1% (Fig. 1b).

To gain functional insight into the identified proteins associated with CD147, we performed functional enrichment analysis using the REACTOME pathway database tool^19^. The analysis revealed that the majority of the proteins were associated with cell surface interactions. For instance, CD147-interacting components included extracellular matrix organization, classical antibody-mediated complement activation, Fc fragment of IgG receptor (FCGR) activation, the role of phospholipids in phagocytosis, and FCGR3A-mediated interleukin 10 synthesis (Fig. 1c). Protein–protein network analysis using STRING database showed that the protein–protein interaction networks were enriched proteins such as CD44, EGFR, CD133, CD276, and MCT4, centered in CD147 in CSCs (Fig. 1d), which aligns with the well-established protein interaction of CD147.

### Co-localization of CD147 proximal proteins in LRM in CSCs

**Figure 2:**
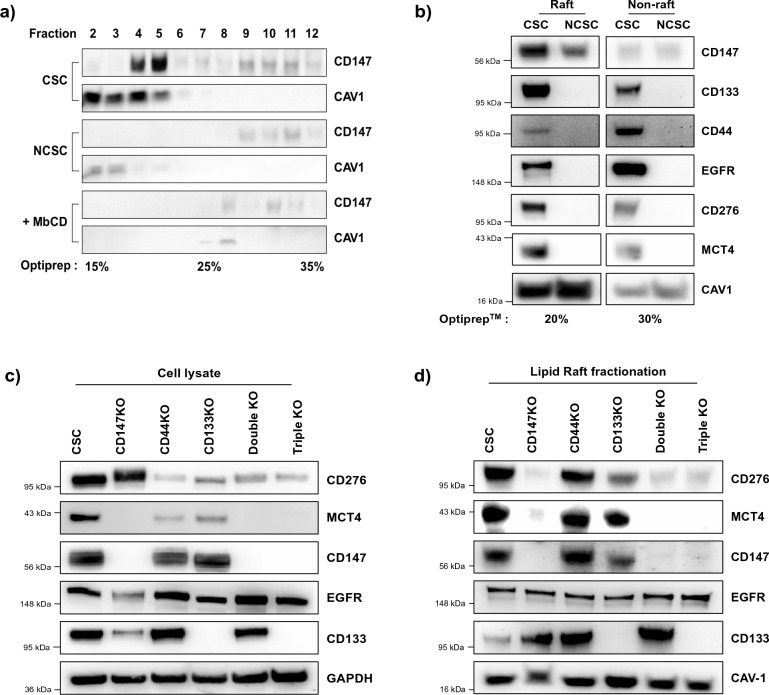
Enrichment and co-localization of CD147 proximal proteins within LRMs. (**a**) Immunoblot analysis showing the enrichment of CD147 in CSCs and NCSCs with and without MbCD treatment. The localization of CD147 to LRMs was determined after fractionation using an Optiprep™ 15–35% gradient. (**b**) Immunoblot analysis demonstrating the co-localization of CD147, CD133, CD44, EGFR, CD276, and MCT4 within the lipid raft fraction (20% Optiprep™) of CSCs and NCSCs. The protein levels were compared to the non-raft fraction (30% Optiprep™). (**c**) Immunoblot analysis of CD276, MCT4, CD147, EGFR, and MCT4 in whole cell lysates prior to lipid raft fractionation. The analysis was performed using CD147KO (CD147 single KO), CD44KO, CD133KO, CD147KO/CD44KO (double KO, CD147 double KO), and CD147KO/CD44KO/CD133KO (triple KO, CD147 triple KO) cell lines. (**d**) Immunoblot analysis of the same proteins in the lipid raft fraction. Full-length blots are presented in Supplementary Figs. S6, S7, S8 and S9.

To gain further insights into the co-localization of CD147 proximal proteins in CSCs, we investigated their localization within LRM. By evaluating lipid raft fractions isolated from a detergent-free density gradient (Optiprep™) ranging from 15 to 35%, we detected CD147 in the 20% Optiprep™ gradient enriched with caveolin 1 (CAV1). Disruption of LRMs with methyl-β-cyclodextrin (MbCD) resulted in the disappearance of CD147 from LRMs, while no CD147 localization was observed in LRMs of NCSCs regardless of MbCD treatment. These findings indicate that CD147 is specifically localized in the CAV1-enriched LRMs in CSCs (Fig. 2a).

Subsequently, we examined the presence of selected proximal proteins (CD147, CD133, CD44, EGFR, CD276, MCT4, and CAV1) in CSCs and NCSCs isolated from the 20% and 30% Optiprep™ fractions, corresponding to the lipid raft and non-lipid raft fractions, respectively. As depicted in Fig. 2b, all the selected proximal proteins, except CAV1, exhibited abundant co-localization within the lipid raft fractions of CSCs but not in NCSCs. Most proteins, except CD44 and EGFR, showed lower abundance in the 30% of non-raft fractions of CSCs. These results suggest that the integrity of lipid rafts is essential for sequestering of CD147 proximal proteins in CSCs.

To investigate whether CD147 influences the assembly of these proteins within LRMs in CSCs, we generated CSC lines with single (CD147, CD44, and CD133), double (CD147/CD44), and triple (CD147/CD44/CD133) target gene knockouts (KOs) (see Supplementary Fig. S2). We examined the changes in the abundance of CD147 proximal proteins within LRMs in CSCs. Our finding indicates that CD276 expression was reduced in the lipid raft fractionation in CD147 single-, double- and triple-KO CSCs. However, CD147 did not affect the surface expression of CD276 expression compared to control cells (CD44KO and CD133KO), as shown in Fig. 2c,d, respectively. Conversely, MCT4 expression was regulated by CD147, as indicated in whole cell lysates, suggesting that CD147 primarily influences the co-localization of CD276 within LRMs rather than promoting trafficking to the cell surface.

### Co-localization of CD147 and CD276 in lipid raft determines the stemness

**Figure 3:**
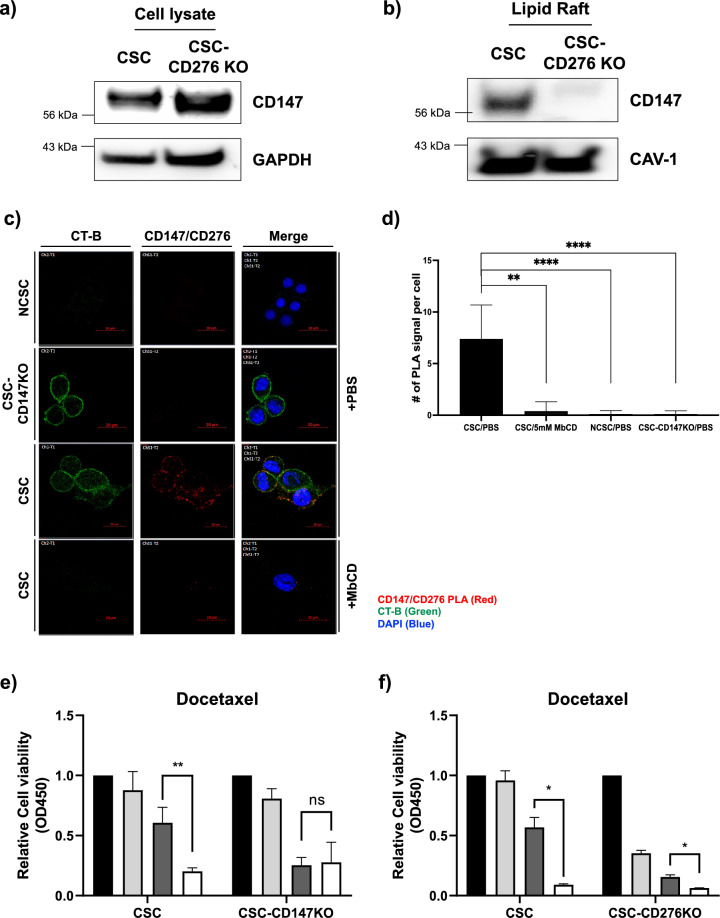
Proximal interaction between CD147 and CD276. Immunoblotting analysis of CD147 expression levels in (**a**) whole lysates, (**b**) the lipid raft fraction of CSC and CSC-CD276 KO cell lines. For full length blots, see Supplementary Fig. S10. (**c**) Double fluorescent microscopic images showing PLA signals (red dots) for the interaction between CD147 and CD276 probed with anti-CD147 and anti-CD276 Abs in NCSCs, CSC-CD147KO, and CSCs. Disruption of the lipid raft in CSC with MbCD treatment (5 mM) reduced PLA signals compared to the controls (+PBS). The lipid raft and the nucleus were stained with the fluorescent marker CT-B (green dots) and DAPI (blue dots), respectively. The overlaid images on the right indicate co-localization of CD147 and CD276 within the LRMs. Scale bars = 20 μm. (**d**) Quantitative analysis of CD147 and CD276 interaction, presented as the number of PLA signals in CSCs, CSCs treated with MbCD, NCSCs, and CSC-CD147KO cells. Values are presented as the mean ± SD from three independent experiments. (**e**) Effects of treatment with docetaxel or co-treatment with docetaxel and MbCD on cell viability of CSCs, compared to CSC-CD147KO and (**f**) CSC-CD276KO cells. Cell viability relative to untreated CSCs was monitored after incubation with docetaxel (25 nM) for 48 h. Data are presented as the mean ± SD (n = 3 for each independent experiment). Statistical significance was determined using Student’s t-tests (*p < 0.05, **p < 0.01, ***p < 0.001, ****p < 0.0001). NS; no significant differences; PLA, proximal ligation assay.

Previous studies have highlighted the role of CD147 in the assembly of various pro-oncogenic proteins, including MCT4, CD44, and CD133 on the plasma membrane, emphasizing its significance in cancer biology^10,20,21^. Consistent with these reports, our study revealed the co-localization of CD276, a co-inhibitory molecule in T-cell-mediated immune responses, within LRMs. To investigate the co-localization between CD147 and CD276, we isolated the lipid raft fractions from the CSC-CD276KO cells and probed them with the anti-CD147 Ab. Immunoblotting of whole-cell lysates from CSC-CD276KO cell lines demonstrated an equal distribution of CD147 (Fig. 3a). However, the expression levels of CD147 within the lipid raft fraction were significantly reduced in CSC-CD276KO cells (Fig. 3b), indicating that CD276 contributes to the sequestration CD147 within LRMs in CSCs.

To further confirm the proximity of CD147 and CD276 within LRMs in CSCs, we employed an *in-situ* proximal ligation assay (PLA). The PLA results, depicted in Fig. 3c, revealed abundant PLA signals (red dots) in CSCs when probed with anti-CD147 and anti-CD276 antibodies, compared to the negative control cells (NCSCs and CSC-CD147KO). Importantly, the specificity of the assay was demonstrated by the lack of non-specific staining in the negative control, as shown in Supplementary Fig. S3, where anti-CD147Ab and anti-CD276Ab were used individually. Conversely, reduced PLA signals were observed in CSC-CD147KO cells and MbCD-treated CSCs. Furthermore, the confocal images overlaying PLA signals with the lipid raft marker CT-B indicated the co-localization of CD147 and CD276 within the LRMs in CSCs (Fig. 3c,d). These findings provide compelling evidence supporting the close association of CD147 and CD276 within the lipid raft microenvironment, which contributes to the stemness phenotype in CSCs.

### CD147 and CD276 in lipid raft promote the stemness phenotype in CSCs

We investigated the role of CD147 and CD276 co-localization in relation to the intrinsic stemness properties of CSCs and their ability to sustain anticancer drug resistance. To assess the impact of CD147 and CD276 on drug resistance, we treated parent CSCs and CSC-CD147KO cells with MbCD alone or in combination with docetaxel (25 nM), and monitored cell viability. As shown in Fig. 3e, MbCD treatment alone did not significantly affect the relative cell viability of both CSCs and CSC-CD147KO cells. However, CSCs exhibited resistance to docetaxel, while CD147KO cells were sensitized to docetaxel, whereas CSCs showed treatment resistance. However, CSC-CD147KO cells became sensitized to docetaxel. Notably, the resistance to docetaxel in CSCs was reversed by MbCD treatment, as evidenced by a similar degree of resistance observed in parental cells and CSC-CD147KO cells (MbCD + Docetaxel). These findings suggest that disrupting the surface assembly of CD147 with MbCD can reverse the resistance phenotype.

Furthermore, we examined the role of CD276, which is proximally co-localized with CD147, in sustaining drug resistance in CSCs. Interestingly, while the viability of CSC-CD276KO cells was affected by MbCD treatment, CSC-CD276KO cells became sensitive to docetaxel (25 nM), whereas CSCs showed treatment resistance. However, the cell viability of CSCs was significantly lower than that of CSC-CD276KO cells upon treatment with docetaxel and MbCD (Fig. 3f). Additionally, our investigation of sphere formation ability revealed a significant decrease in the number and size of spheres formed by CSCs with CD276KO, CD147KO, and MbCD treatment compared to the control group (Supplementary Fig. S4). This finding reinforces the importance of the lateral interactions between CD276 and CD147 within lipid rafts for the maintenance of stemness in BC.

**Figure 4:**
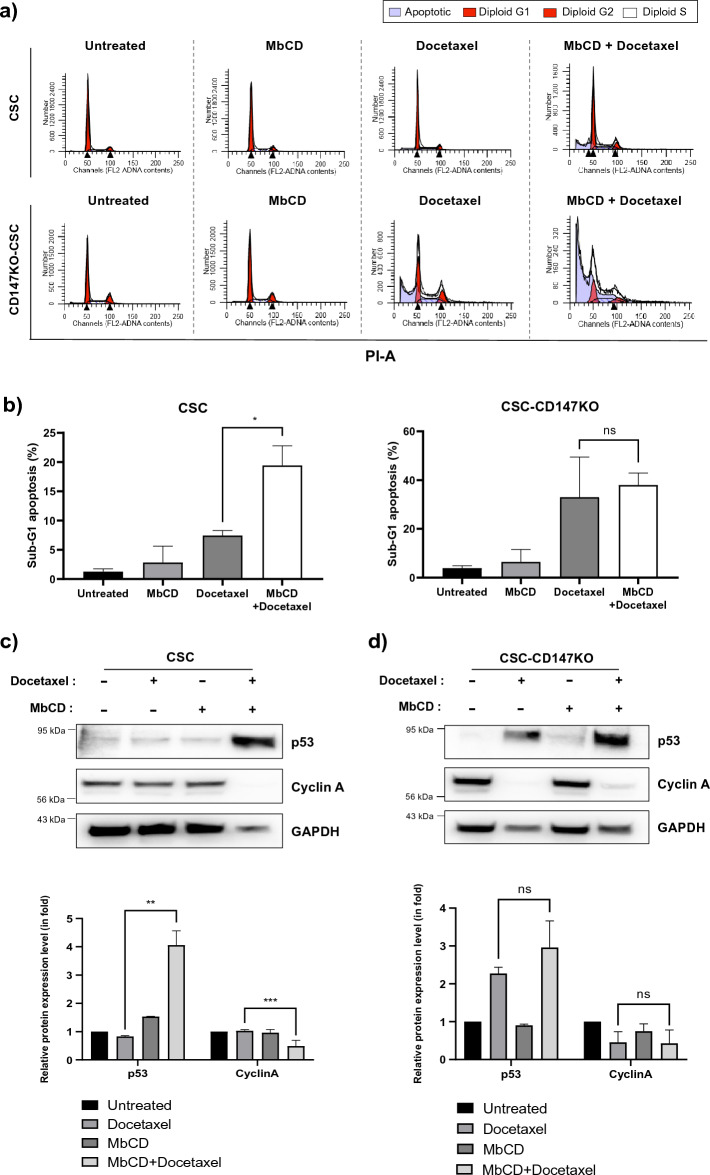
Effect of docetaxel on cell cycle progression and protein expression in CSCs. (**a**) FACS analysis of cell cycle distribution in CSCs treated with docetaxel alone or co-treated with docetaxel and MbCD, compared to CD147KO CSCs. (**b**) Quantification of the sub-G1 phase, indicating apoptosis, in CSCs after treatment with docetaxel or co-treatment with docetaxel and MbCD, relative to CD147KO CSCs. Western blotting analysis and quantification of cell cycle-related proteins (cyclin A and P53) (**c**) in CSCs treated with docetaxel alone or co-treated with docetaxel and MbCD in comparison (**d**) with CD147KO CSCs. Quantification was performed using densitometric analysis and normalized to GAPDH. Full-length blots are presented in Supplementary Fig. S11. Statistical significance was determined by the student's t-test (*p < 0.05).

We further investigated the effects of docetaxel treatments on the cell cycle distribution of the CSCs and CSC-CD147KO cells, which are associated with the unique features of cell-surface assembly. Flow cytometry analysis revealed a higher percentage of the sub-G1 fraction, indicating apoptotic cells. In CSCs treated with docetaxel and MbCD compared to cells treated with docetaxel alone or the control group (untreated). The increase in sub-G1 arrested cells was observed in CSC-CD147KO cells treated with docetaxel or docetaxel and MbCD, indicating that the resistance to docetaxel was lost in CSC-CD147KO cells regardless of the disruption of surface assembly caused by the docetaxel/MbCD treatment (Fig. 4a bottom panel and 4b).

To investigate the mechanism underlying the cell cycle arrest induced by the disruption of cell-surface protein assembly in CSCs, we analyzed the levels of regulatory proteins, p53 and cyclin A, by immunoblotting in both CSCs and CSC-CD147KO cells. In CSCs, the level of p53 significantly increased, while cyclin A level was markedly decreased compared to the control group or the MbCD-only treatment and the docetaxel-only treatment (Fig. 4c). In CSC-CD147KO cells, the levels of p53 increased after docetaxel-only treatment or docetaxel/MbCD co-treatment, accompanied by a decrease cyclin A level (Fig. 4d). These results indicate that the docetaxel-induced cell cycle arrest in CSCs depends on the co-localization of CD147 proximal proteins.

### Clinical significance of CD147 and CD276 co-expression in chemotherapy resistance in HER2+ BC and TNBC

**Figure 5:**
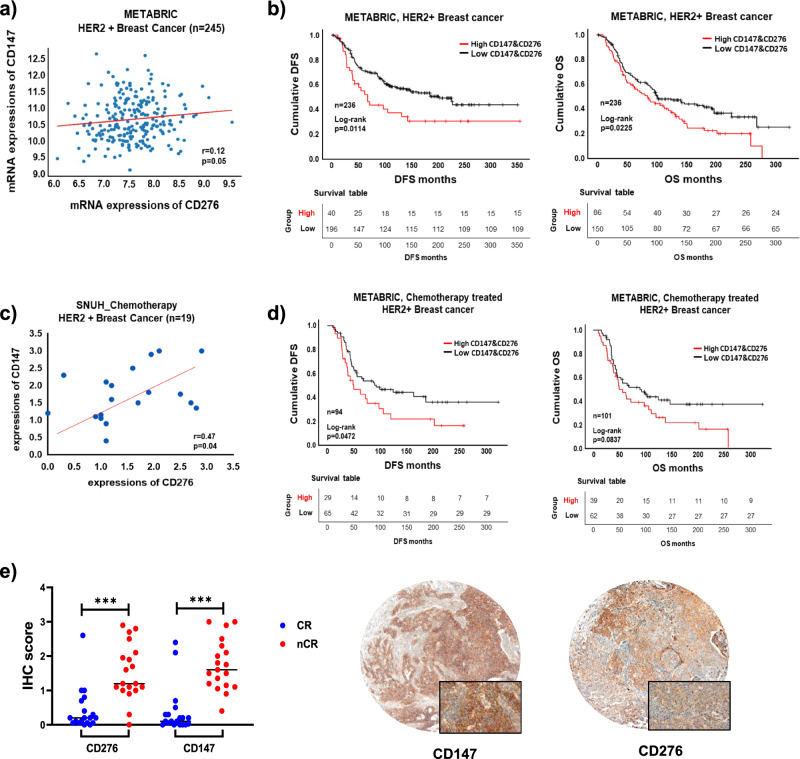
Co-expression of CD147 and CD276, and its clinical impact in patients with HER2+ BC who received chemotherapy. (**a**) Scatterplot showing the correlation between CD147 and CD276 in HER2+ BC patients, regardless of chemotherapy use, from the METABRIC dataset. (**b**) Disease-free survival (DFS) and overall survival (OS) based on combined CD147 and CD276 mRNA expression in a cohort of 236 HER2+ BC cases from the METABRIC dataset. (**c**) Scatterplot demonstrating the correlation between CD147 and CD276 mRNA expression in HER2+ BC patients who received chemotherapy in our neoadjuvant chemotherapy cohort. (**d**) DFS and OS analysis of the HER2+ BC cohort treated with chemotherapy based on the METABRIC dataset. (**e**) Scatterplot illustrating complete response (CR) to chemotherapy in HER2+ BC patients from our neoadjuvant chemotherapy cohort (Left, CR: complete response; nCR: non-complete response) and representative images of immunohistochemical analysis images of CD147 and CD276 in HER2+ BC cases from our cohort (Right, original magnification × 40 and × 400 [inlet], respectively).

To determine the clinical relevance of CD147 and CD276 co-expression in BC, we analyzed their expression profiles using the Molecular Taxonomy of Breast Cancer International Consortium (METABRIC) database. We observed a weak but a positive correlation between CD147 and CD276 mRNA expression in HER2+ BC (ρ = 0.12, *p* = 0.05) (Fig. 5a). We further investigated the association between CD147 and CD276 expression and clinicopathological characteristics (Fig. 5b). Concurrent mRNA expression of CD147 and CD276 in HER2+ BC patients was associated with poor disease-free survival (DFS) and overall survival (OS) rates (*p* = 0.01 and 0.02, respectively) (Fig. 5b). Similar findings were observed in HER2+ BC patients who received chemotherapy. In our in-house patient cohort, elevated mRNA expression of CD147 and CD276 showed a significant correlation with each other (ρ = 0.47 and *p* = 0.04, respectively) (Fig. 5c). The METABRIC database also revealed that higher concurrent expression of CD147 and CD276 was associated with unfavorable survival outcomes in HER2+ BC patients who received chemotherapy, both for DFS and OS (*p* = 0.04 and 0.08, respectively) (Fig. 5d). Moreover, in an independent HER2+ BC cohort, higher expressions of CD147 or CD276 were associated with a lower complete response to chemotherapy (CD147 and CD276: *p* < 0.01) (Fig. 5e). These findings provide clinical evidence supporting the significance of CD147 and CD276 dual expressions in mediating chemotherapeutic resistance in HER2+ BC. Furthermore, we further extended our analysis to include a TNBC patient cohort to assess the clinical significance of CD147 and CD276 co-expression in this BC subtype. In TNBC, we found that the dual mRNA expression of CD147 and CD276 was associated with unfavorable disease-free survival (DFS) and overall survival (OS) rates (*p* = 0.02 and 0.03, respectively) (Supplementary Fig. S5A). We also examined the impact of CD147 and CD276 co-expression on prognosis in TNBC patients who received chemotherapy and consistently observed significantly reduced OS (*p* = 0.0139) and DFS (*p* = 0.0103) associated with higher co-expression of CD147 and CD276 (Supplementary Fig. S5B).

These findings demonstrate that the co-expression of CD147 and CD276 is not limited to the HER2+ BC population but also holds prognostic significance in TNBC. The association between CD147 and CD276 expression and adverse clinical outcomes in TNBC patients provides insights into the potential role of these markers in cancer stemness and drug resistance.

## Discussion

In cancer cells, lateral interactions among assembled oncogenic membrane proteins within spatially restricted intracellular compartments play a crucial role in promoting aggressive phenotypes^1^. Therefore, understanding the detailed analysis of these oncogenic protein complexes can provide mechanistic insights into cellular events at the plasma membrane level. In this study, we focused on analyzing the lateral interactions of oncogenic membrane proteins in BC, specifically examining the CD147 co-assembled proteins using a proximal labeling approach. Our aim to elucidate the integrity of the cell-surface phenotype of CSCs associated with BC cell stemness. Furthermore, we sought to validate the co-expression of CD147 and CD276 in BC patients and determine clinical relevance.

Previous studies have demonstrated the involvement of CD147 in BC invasion, proliferation, and drug resistance^22^. High expression of CD147 is associated with tumor invasion and metastasis and it forms complexes with CD44 and EGFR in the LRMs, promoting invasiveness in breast epithelial cells through the hyaluronan-CD44-dependent EGFR-Ras ERK signaling pathway^9^. Perturbation of the interaction between CD147 and CD44 leads to the suppression of lactate efflux, affecting the glycolytic phenotype of BC cells^10,23^. CD147 has also been implicated in chemoresistance BC CSCs^7,24^, including the regulation of cancer stemness.

In our study, we utilized CSCs derived from OCT3/4-enriched MDA-MB453 cells^12^ to investigate the cell-surface phenotype associated with CD147. Using proximity proteomics^18^, We identified several CD147 putative proximal proteins including CD44, EGFR, CD133, MCT4, and integrin family proteins, which are known to be involved in regulating cancer stemness^25,26^. These proteins were found to be localized in LRMs in CSCs, supporting previous findings on the association between MCT4 and CD147. MCT4 co-localized with CD147 to the surface of a highly metastatic breast cancer MDA-MB231 cell line is associated with poor prognosis in prostate cancer and other cancer types^20,27,28^.

Furthermore, we identified CD276 as a CD147 proximal protein in CSCs. CD276 is aberrantly overexpressed in numerous cancer types and is associated with poor clinical prognosis^29^. In breast CSCs, which exhibit high levels of CD147, we observed markedly higher expression of CD276 compared to other B7 family proteins, such as PD-L1 and CTLA-4^7^. However, the molecular functions of CD276 in BC CSCs remain poorly understood. Our study revealed that the lateral interaction between CD147 and CD276, sequestered within the LRMs is a unique cell-surface phenotypic feature of CSCs. Immunostaining, disruption of the LRMs, and PLA data support the existence of proximal interactions between CD147 and CD276. In addition to its immune regulatory function of CD276, our findings suggest a novel functional role of CD276 in anticancer drug resistance, particularly in CSCs.

To validate the clinical relevance of CD147 and CD276 co-expression, we analyzed tissue samples from BC patients. We found a significant correlation between the co-expression of CD147 and CD276 and its clinical impact in HER2+ BC and TNBC. Higher expression of CD147 and CD276 in patients with HER2+ BC and TNBC who received chemotherapy was associated with a lower complete response to chemotherapy, as supported by in independent cohorts of HER2+ BC and TNBC patients. These results provide further support for the clinical significance of dual CD147 and CD276 expression, which mediates chemotherapeutic resistance in HER2+ BC and TNBC.

Overall, our study highlights the importance of lateral interactions among oncogenic membrane proteins, particularly CD147 and CD276, in driving aggressive phenotypes and drug resistance in BC. The co-localization of CD147 and CD276 in LRMs, their impact on cancer stemness, and their association with unfavorable clinical outcomes and chemotherapeutic resistance in HER2+ BC and TNBC provide valuable insights into the potential therapeutic targeting of these proteins.

## Conclusion

This study provides novel insights into the cell-surface assembly of oncogenic proteins involving CD147 and its association with CD276 in breast CSCs. We demonstrate that the co-expression of CD147 and CD276 in BC CSCs is associated with cancer stemness and drug resistance, in addition to its known immunological function. Furthermore, our analysis of BC tissue samples reveals that the co-expression of CD147 and CD276 may serve as a prognostic indicator for poor outcomes in patients with BC. This is the first study to investigate the significance of the lateral interaction between CD147 and CD276 in CSCs, shedding light on the functional role of CD147 and its proximal proteins in cancer cell stemness. These findings contribute to a better understanding of the mechanisms underlying aggressive phenotypes and therapeutic resistance in BC and may have implications for the development of targeted therapies against CD147 and CD276 in the context of BC. Further studies are warranted to explore the therapeutic potential of targeting CD147 and its associated proteins to overcome drug resistance and improve patient outcomes in BC.

## Material and methods

### Cell culture and reagents

CSC-like cells and NCSC-like cells derived from the MDA-MB453 were provided by the University of Pittsburgh Medical Center (Pittsburgh, PA, USA)^12^. The cell lines were cultured using Dulbecco's modified Eagle’s medium containing 10% fetal bovine serum and 1% penicillin/streptomycin at 37 °C with 5% CO_2_. Acetonitrile (ACN), formic acid (FA), urea, dithiothreitol, iodoacetamide, and ammonium bicarbonate were purchased from Sigma–Aldrich (St. Louis, MO, USA). Sep-Pak C18 cartridges were obtained from Waters Corporation (Milford, MA, USA). Trypsin protease MS-grade was purchased from Thermo Scientific (Rockford, IL, USA). Antibodies against CD147 and CD44 were developed in-house^30^, unless stated otherwise. GAPDH and CD133 were purchased from Cell Signaling Technology (Danvers, MA, USA) and Genetex (Irvine, CA, USA), respectively. CD276 and MCT4 Abs were obtained from R&D Systems (Minneapolis, MN, USA) and Santa Cruz Biotechnology (Dallas, TX, USA), respectively. Secondary antibodies were purchased from AbFrontier (YoungIn Frontier, Seoul, Korea) and Jackson ImmunoResearch Laboratories (West Grove, PA, USA).

### Proximity labeling of CD147 proximal proteins by anti-CD147-HRP

Cleavable tyramide-biotin was prepared by mixing 5 mg of EZ-Link™-biotin (Thermo Scientific) with 1.55 mg of tyramide hydrochloride (Sigma–Aldrich) in 100 µL of dimethyl sulfoxide in 2 mL of PBS. The mixture was incubated overnight at room temperature in the dark. After filtration, the tyramide-biotin was stored at − 20 °C until use. HRP was conjugated to anti-CD147 and huIgG using the EZ-Link™ Plus Activated Peroxidase kit (Thermo Scientific) according to the manufacturer’s instructions. For proximity labeling, cells (2 × 10^7^) were washed with ice-cold PBS and incubated with anti-CD147-HRP Ab or huIgG-HRP (negative control) for 2 h at 4 °C. Following incubation, the cells were washed with ice-cold PBS and biotinylated for 15 min with tyramide-labeled buffer. After removal of unreacted reagents, the cells were lysed using RIPA lysis buffer (Thermo Scientific) via sonication on ice. Protein concentration was determined using the bicinchoninic acid (BCA) Protein Assay Kit (Thermo Scientific). Biotin-labeled CD147 proximal proteins by anti-CD147-HRP were isolated using streptavidin magnetic beads (Thermo Scientific) according to the manufacturer’s instructions.

### SDS-PAGE fractionation and in-gel digestion

Protein samples (100 µg) were fractionated on 4–12% Bis–Tris Gels (Invitrogen, Carlsbad, CA, USA) and stained using Instant Blue (Sigma–Aldrich). In-gel digestion was performed following the general protocol^31^. Briefly, the protein bands were cut, destained, and washed. Proteins were reduced using 20 mM dithiothreitol for 1 h at 60 °C and alkylated with 55 mM iodoacetamide at room temperature for 45 min in the dark. After dehydration, the proteins were digested with 13 ng/µL sequencing-grade modified porcine trypsin (Promega Corporation, Madison, WI, USA) in 50 mM NH_4_HCO_3_ overnight at 37 °C. Peptides were extracted from the gel slices using 50% v/v ACN in 0.1% v/v FA, and 80% v/v ACN in 0.1% v/v FA and dried under vacuum.

### MS and protein database search

Peptides were resuspended in 20 μL solvent A (0.1% FA in water), and 9 μL of the sample was injected through a trap column (PepMap™ RSLC C18 column 75 μm ID*2 cm, 3 μm, Thermo Fisher Scientific) fused with an analytic column (PepMap™ RSLC C18 column 75 μm ID*50 cm, 2 μm, Thermo Fisher Scientific) and separated using a linear gradient of 5%–35% solvent B (0.1% FA in ACN) for 90 min at a flow rate 300 nL/min. Samples were analyzed in duplicate on a Q-Exactive (Thermo Fisher Scientific) hybrid quadrupole-Orbitrap MS, interfaced with a high-performance LC system. The spray voltage was set to 2.2 kV, and the temperature of the heated capillary was set to 250 °C. The full scans were acquired using the mass analyzer at 400–1,400 m*/z* with a resolution of 70,000. The MS/MS scans were obtained at a resolution of 17,500 using normalized collision energy of 27 eV for high-energy collisional dissociation fragmentation. The advanced gain control target and maximum injection time were 5 × 10^4^ and 120 ms, respectively. The isolation window was set at 3 m*/z*. The Q-Exactive was operated in the data-dependent mode with one survey MS scan followed by 10 MS/MS scans, and the duration of dynamic exclusion was 60 s. Collected MS/MS data were converted into mzXML files using the Trans Proteomic Pipeline (version 4.5) software and searched against the decoy Uniprot Human database (version 3.83, 186 578 entries) to estimate the false discovery rate using the SEQUEST® (version 27; Thermo Fisher Scientific) program in the Peaks Studio ver. 8.5 (Bioinformatics Solutions, Inc., Waterloo, ON, Canada) database search platform. Precursor and fragment ion tolerance were set to 10 ppm and 0.5 Da, respectively. Trypsin was selected as the enzyme with a maximum allowance of up to two missed cleavages. Carbamidomethyl of cysteine and oxidized methionine were set as fixed and differential modification search, respectively. The Scaffold software package (version 3.4.9; Proteome Software Inc., Portland, OR, USA) was used to validate MS/MS-based peptide and protein identification. Peptide and protein identification were accepted if they could be established at > 95% and > 99% probability, respectively, as specified by the Peptide and Protein Prophet algorithm^32,33^, and if the protein identification had at least two identified peptides with a false discovery rate < 0.1%. Spectral counting-based semi-quantitative proteome analysis was used to determine relative changes in the abundance of proteins. The MS/MS data were normalized to compare protein abundances between samples using the Scaffold software (version 3.4.9; Proteome Software Inc.). Statistically significant changes in proteins between two cells were determined by comparing normalized spectral numbers via duplicate analysis using the R program and power law global error model^34^. The subcellular localization and functional annotation of the identified proteins were categorized using the Ingenuity Pathway Analysis (Ingenuity Systems) and PANTHER (Protein Analysis Through Evolutionary Relationships) Classification System (v7.2). Protein–protein interactions and networks of the identified proteins were annotated using Ingenuity Pathway Analysis. The mass spectrometry proteomics data have been deposited to the ProteomeXchange Consortium^35^ via the PRIDE partner repository with the dataset identifier PXD036909.

### Generation of stable CSC target gene knockout cell lines

The CSCs containing a single knock out of target genes CD147, CD44, CD133, and CD276 were generated using the CRISPR/Cas9 gene-editing system. Briefly, CD147 or CD44-specific single guide RNA (sgRNA), which was designed using CHOPCHOP^36^, was ligated to the linearized pGuide-it-tdTomato vector (Takara, Kusatsu, Japan) and then co-expressed with the Cas9 nuclease and fluorescent protein tdTomato. The CD133 or CD276-specific sgRNA was cloned into the pSpCas9(BB)-2A-Puro (PX459) V2.0 vector (Addgene, MA, USA) via the BbsI restriction site to generate the CSC-CD133KO and CSC-CD276KO cells. The construction of the plasmid was confirmed by DNA sequencing. The CSC-MDA-MB453 cells (1 × 10^6^) were transfected with 10 μg target-specific sgRNA/pGuide-it-tdTomato or sgRNA/PX459 plasmid by electroporation. Following 2–3 days of transfection, individual CSC-CD147KO and CSC-CD44KO clones were selected by screening for tdTomato-positive CSC cells using fluorescence microscopy. The clones were then expanded to generate stable KO cell lines. The CSC-CD133KO and CSC-CD276KO cells were selected by identifying puromycin-resistant clones in DMEM growth medium supplemented with 10% FBS and 250 ng/mL puromycin; during the selection, the selective medium was replaced every 3–4 days.

The CD147/CD44 double-knockout CSCs were generated by transfecting stable CSC-CD44KO cells with the CD147-specific sgRNA/pGuide-it-tdTomato Cas9 vector. The stable double knockout of CD147 and CD44 in CSCs (CSC-CD147/CD44 DKOs) was identified by screening for tdTomato-positive cells using fluorescence microscopy and sorting of tdTomato-positive cells that were negative for CD147 and CD44 during FACS analysis with anti-CD147 and anti-CD44 antibody. Similarly, the CSCs with the stable triple-knockout (TKO) of CD147, CD44, and CD133 were generated by transfection of CSC-CD147/CD44DKO with the CD133 sgRNA/PX459 Cas9 vector. The CSC-CD147/CD44/CD133 TKO cells were selected in the selective medium with puromycin, followed by cell sorting by flow cytometry using FACSAria II Flow Cytometer (BD Biosciences) at the Central Laboratory of Kangwon National University. The clones with target gene(s) knock out were confirmed by FACS using the corresponding antibody against the target protein (see Supplementary Fig. S2).

### Isolation of lipid rafts microdomains

Membrane lipid rafts were isolated according to the protocol described by Macdonald and Pike^37^. Briefly, the cells were scraped in 1 mL of lysis buffer (10 mM Tris–HCl, 1 mM EDTA, 200 mM sucrose, Roche protease inhibitor cocktail, pH 7.4) and centrifuged at 250 × *g* for 2 min. After the addition of Triton X-100 (1% v/v), the cell lysate was mechanically disrupted by probe sonication for 5 min, incubated on ice for 30 min, and centrifuged at 14,000 × *g* for 30 min. The cell lysates were directly mixed with iodixanol stock solution (60% solution of Optiprep iodixanol) to yield a 40% (v/v) iodixanol-lysate solution, which was placed at the bottom of an ultracentrifuge tube. Equal volumes of 0%–20% Opti-prep in lysis buffer without Triton X-100 were carefully overlaid above the iodixanol-lysate solution. The samples were centrifuged at 200,000 × *g* for 4 h at 4 °C, and fractions of 1 mL (typically 11–12 fractions in total) were collected from the top of the density gradient tube.

### Immunoblotting

Immunoblotting was performed following the general protocol. Briefly, whole lysates were obtained using the RIPA lysis buffer, and the amount of the protein in the samples was quantified using the BCA assay. Proteins were resolved by SDS-PAGE and transferred to polyvinylidene difluoride. The membrane was blocked with 5% skim milk or 3% bovine serum albumin in tris-buffered saline containing 0.1% Tween-20 and probed with appropriate primary antibodies. HRP-linked secondary antibodies bound to the primary antibodies were detected using enhanced chemiluminescence. The signal intensity was quantified by Multi-Gauge software (Fujifilm).

### Cell viability assay

According to the manufacturer’s instructions, cell viability was evaluated using the water-soluble tetrazolium salt assay using the EZ-Cytox assay kit (Wellbio, Seoul, Korea). Cells were seeded in 96-well plates at 5 × 10^4^ cells/mL. For analysis of cell viability, 10 μL of water-soluble tetrazolium salt reagent solution was added to each well, and the reaction was allowed to proceed for 1 h at 37 °C. Absorbance was measured at 450 nm using a microplate reader.

### Immunofluorescence imaging

Cells grown on 35 mm confocal imaging dish (SPL, South Korea) were incubated with HRP-conjugated anti-CD147 antibody for 1 h at 4 °C and treated with tyramide-labeling buffer (50 mM Tris–HCl, pH 7.4, with fresh 0.03% H_2_O_2_ containing 40 µg/mL tyramide-biotin label) for 15 min at room temperature. Cells were stained with 2 µg/ml of SA-TexasRed to detect biotin and 5 µg/ml of Alexa-647 conjugated Goat anti-human IgG-Fc to detect anti-CD147 antibody for 1 h on wet ice. Cells were washed twice with PBS and fixed with 4% paraformaldehyde for 20 min at room temperature. The nuclei were stained with DAPI.

### Proximity ligation assay (PLA)

PLA was performed using Duolink II Detection Kit to confirm protein–protein interactions according to the manufacturer’s instructions (Sigma–Aldrich). Briefly, blocked cells were incubated with anti-CD147 and anti-CD276 antibodies for 1 h on wet ice. After washing with PBS, the lipid rafts of cells were stained with 10 ug/mL of Cholera Toxin Subunit B (CT-B) for 30 min at 4 °C. The cells were fixed with 4% paraformaldehyde for 20 min at room temperature and then incubated with PLA probes (oligonucleotides) for 1 h at 37 °C. After washing with PBS, cells were ligated for 30 min at 37 °C. Subsequent amplification with polymerase was performed for 150 min at 37 °C. Nuclei were stained with DAPI. Red fluorescent signals from the PLA were visualized using a confocal laser scanning microscope (LSM 880; Carl Zeiss, Jena, Germany). The number of red dots of each cell was quantified using Image J software (NIH, Bethesda, MD, USA), where 100 cells of each group were analyzed in at least three independent experiments.

### Cell cycle analysis

Cells were detached using 0.1% trypsin in 2.5 mM EDTA and washed with ice-cold PBS. Subsequently, the cells were fixed with 66% (v/v) ethanol on ice for a least 30 min and washed with ice-cold PBS. Next, the cells were digested using RNase A (500 U/mL) for 30 min at 37 °C and stained with propidium iodide (50 µg/mL). The DNA content (10,000 cells per experimental group) was determined using a FACS Calibur flow cytometer (Becton Dickinson Biosciences, San Jose, CA, USA) equipped with a ModFit LT program (Verity Software House, Topsham, ME, USA).

### Tumor sphere formation assay

Spheroid cultures were generated by seeding a defined number of cells onto 24-well Black/Clear Round Bottom Ultra-Low Attachment (Corning) in 1 mL of cell culture medium. After seeding, the Ultra-Low Attachment plates were centrifuged at 100 × *g* for 3 min, to allow for cell aggregation, and cultured at 37 °C and 5% CO_2_ atmosphere. The spheroid formation derived from CSCs, CSC-CD147KO cell lines and CSC-CD276KO cell lines with or without MbCD treatment was performed. Over an incubation period of nine days, spheroids were imaged and captured using a Nikon Eclipse microscope (Nikon, Japan). For cell death, spheroid formation cells were counted by the trypan blue exclusion assay.

### Ethics approval

The study protocol was approved by the Institutional Review Board (IRB) of Seoul National University (IRB number 2103/001-014) under the premise that the anonymity of the patient is guaranteed. In consideration of the large number of patients and the fact that this is a retrospective study, obtaining informed consent was exempted by the IRB of Seoul National University. Our research involving human data has been performed in accordance with the Declaration of Helsinki.

### Patient and clinical tissue sample selection

All pathologic specimens enrolled in this study were collected from the Seoul National University Hospital biorepository operated by the Department of Pathology. The inclusion criteria were: (1) female patients who received chemotherapeutic medication with doxorubicin, cyclophosphamide, and docetaxel as preoperative therapy^38^; (2) biopsy samples available per-chemotherapeutic needle and post-chemotherapeutic surgical specimens for microscopic assessment of therapeutic effectiveness by the American Joint Committee on Cancer TNM^39^; (3) histologically confirmed T2 tumors with positive axillary nodes (N1); and (4) available immunohistochemical information for intrinsic subtyping. Tissue samples for immunohistochemical validation were divided into the following two sets: a discovery set consisting of a total of 38 needle biopsy samples collected from patients with non-pathologic and pathologic complete response (19 patients each).

### Survival analysis with public data

The associations between CD147 and CD276, and DFS and OS in HER2+ human BC and TNBC were analyzed using the METABRIC and GSE19615 (RNA‐Seq data: Gene Expression Omnibus) databases. Survival analysis was performed separately in the HER2+ BC and TNBC dataset based on chemotherapy. The DFS and OS of patients with BC in METABRIC and gene set enrichment datasets were analyzed through Cancer Target Gene Screening (http://ctgs.biohackers.net)^40^. A publicly available dataset from human breast cancer patients, METABRIC, was downloaded from cBioPortal (http://www.cbioportal.org/) and re-analyzed to investigate correlations and survival analyses.

### Immunohistochemistry

Immunohistochemical staining was performed in formalin-fixed paraffin-embedded samples from 38 cases of HER2+ human BC obtained through needle biopsy prior to chemotherapy. The blocks were sectioned (thickness: 3 μm), and standard immunohistochemistry procedures for the slides prepared by fixation in 10% neutral buffered formalin solution or 95% ethanol were performed using a BenchMark automatic immunostaining device (Ventana BenchMark XT Staining System, Tucson, AZ, USA). Slides were incubated with anti-CD147 (GTX20666; GeneTex; dilution 1:75) and anti-CD276 (AF1027; R&D Systems; dilution 1:2,000). The immunohistochemical interpretation was evaluated by a semi-quantitative approach using an “H-score”^41^ in blind and independent manner by two pathologists (HSR and MSJ).

### Statistical analysis

All statistical analyses were performed using GraphPad Prism 8 (GraphPad, San Diego, CA, USA). Results are presented as the means ± standard error of the means. Differences between two groups were assessed by the Student t-test. The error bars represent ± standard deviation of the experiments. We filtered candidate proteins for immunohistochemical validation using the Mann–Whitney U test to obtain variables showing significant differences between the two groups.

### Supplementary Information

Supplementary Figures.

## Data Availability

The mass spectrometry proteomics data have been deposited to the ProteomeXchange Consortium via the PRIDE partner repository with the dataset identifier PXD036909. The other data that support the findings of this study are publicly available from the corresponding author upon reasonable request and can be down.
