# Supplementary material for: Lateral interactions between CD276 and CD147 are essential for stemness in breast cancer: a novel insight from proximal proteome analysis
Source: Sci Rep. 2023 Aug 30;13:14242. doi: 10.1038/s41598-023-41416-7 (PMC10469185; doi:10.1038/s41598-023-41416-7)
Supplement: Supplementary file 1 — Supplementary Figures. [file 41598_2023_41416_MOESM1_ESM.docx]

Supplementary Information

**Supplementary Figures**

**Supplementary Figure S1.** Outline of experimental workflow employed to identify the CD147 proximal proteome.

**Supplementary Figure S2.** Verification of target gene knockout (KO) in CSCs.

**Supplementary Figure S3.** Proximal interaction between CD147 and CD276

**Supplementary Figure S4.** Tumorsphere formation assays in CD147 knock-out (CSC-CD147KO), CD276 knock-out (CSC-CD276KO), and methyl-β-cyclodextrin (MbCD)-treated CSCs.

**Supplementary Figure S5.** Co-expression of CD147 and CD276, and its clinical impact in patients with TNBC undergoing chemotherapy.

**Supplementary Figure S6.** Uncropped immunoblot images corresponding to Figure 2a.

**Supplementary Figure S7.** Uncropped immunoblot images corresponding to Figure 2b.

**Supplementary Figure S8.** Uncropped immunoblot images corresponding to Figure 2c.

**Supplementary Figure S9.** Uncropped immunoblot images corresponding to Figure 2d.

**Supplementary Figure S10.** Uncropped immunoblot images corresponding to Figure 3a and b

**Supplementary Figure S11.** Uncropped immunoblot images corresponding to Figure 4c and d.

**
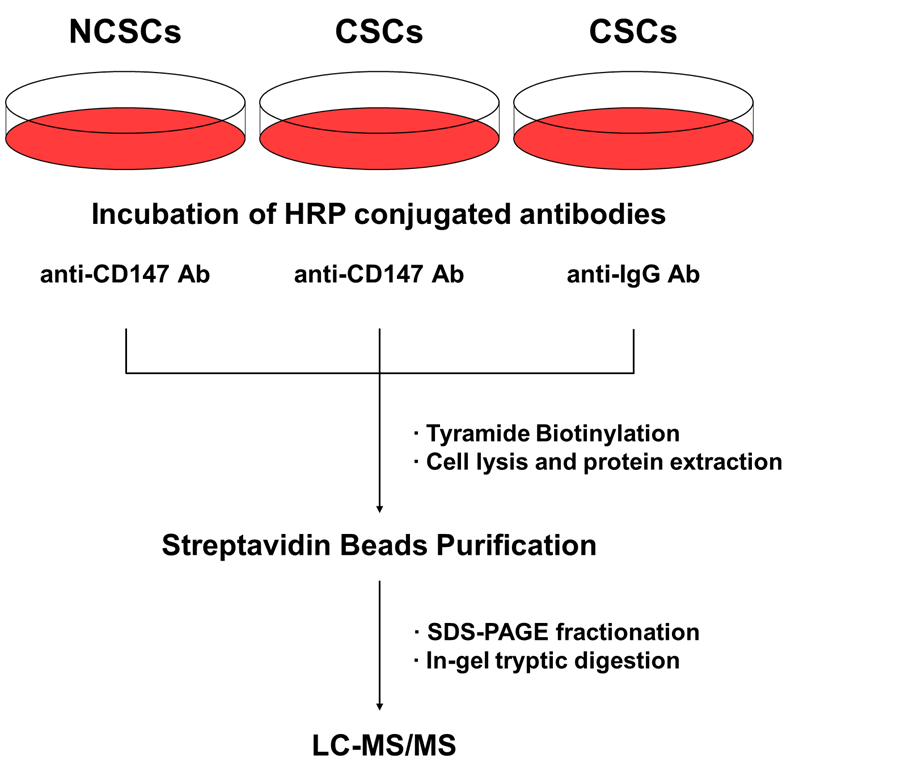
**

**Supplementary Figure S1. Outline of experimental workflow employed to identify the CD147 proximal proteome.** The procedure involves incubation of anti-CD147-HRP with hydrogen peroxide and biotin-tyramide, which generates radicals that covalently label the proteins in the vicinity of CD147. The biotinylated proteins are then isolated through streptavidin purification, followed by in-gel tryptic digestion and subsequent LC-MS/MS analysis.

**Supplementary Figure S2. Verification of target gene knockout (KO) in CSCs**. The protein expression patterns of CD147, CD44, CD133, and CD276 were assessed using (**A**) Western blotting and (**B**) flow cytometry analysis. The protein expression profiles of cells with single target gene KO, double KO, and triple KO were compared to the wild-type (WT) cells. Further details regarding the experimental procedures can be found in the main text.

**Supplementary Figure S3. Proximal interaction between CD147 and CD276**

CSCs, NCSCs, and CSC-CD147KO cells were cultured under the indicated conditions. The co-localization of CD147 with other proteins in cultured cells was analyzed using proximity ligation assay (PLA), represented by red dots. Nuclei were stained with DAPI (blue).

In panels (**A-D**), the cross-linking of the anti-CD147 antibody resulted in increased CD147/CD276 interaction in CSCs treated with PBS but not in those treated with 5 mM MbCD, NCSCs, and CSC-CD147KO cells. Negative control experiments using (**E**) anti-CD147 and (**F**) anti-CD276 antibodies did not show any interactions. The scale bars indicate 20 μm.

**Supplementary Figure S4. Tumorsphere formation assays in CD147 knock-out (CSC-CD147KO), CD276 knock-out (CSC-CD276KO), and methyl-β-cyclodextrin (MbCD)-treated CSCs.**

The representative image displays tumorspheres derived from CSC-CD147KO, CSC-CD276KO, and CSCs after treatment with the indicated concentrations of MbCD for 4 days (scale bar: 100 μm). Additionally, statistical analysis presents the number of tumorspheres (% of Control) in CSC-CD147KO, CSC-CD276KO, and CSCs treated with MbCD for 4 days. The tumorsphere numbers are presented as the mean ± SD from three replicated wells. Statistical significance is denoted as ****p* <0.001 and *****p* <0.0001 compared to the control.

**Supplementary Figure S5. Co-expression of CD147 and CD276, and its clinical impact in patients with TNBC undergoing chemotherapy.** (**A**) the disease-free survival (DFS) and overall survival (OS) outcomes are assessed in relation to the combined mRNA expression of CD147 and CD276 among TNBC cases from the METABRIC dataset. This analysis provides insights into the prognostic significance of CD147 and CD276 co-expression in TNBC patients. (**B**) the DFS and OS analysis focuses on the TNBC cohort specifically treated with chemotherapy from the METABRIC dataset.

**Supplementary Figure S6. Uncropped immunoblot images corresponding to Figure 2a.** Chemiluminescent images are merged with brightfield images of the membranes to aid in the visualization of protein markers. Blue boxes highlight bands that were cropped for representative images in the main text. The red box indicates the band that serves as the loading control, as mentioned in the main text. Blots underwent trimming before hybridization with antibodies.

**Supplementary Figure S7. Uncropped immunoblot images corresponding to Figure 2b.** Chemiluminescent images are merged with brightfield images of the membranes to aid in the visualization of protein markers. The bands on the membranes represent the target of (**A**) CD147, (**B**) CD133, (**C**) CD44, (**D**) EGFR, (**E**) CD276, and (**F**) MCT4. Blue boxes indicate the bands that were cropped for representative images shown in the main text. The red box indicates the band that serves as the loading control, as mentioned in the main text.

**Supplementary Figure S8. Uncropped immunoblot images corresponding to Figure 2c.** Chemiluminescent images are merged with brightfield images of membranes to aid in the visualization of protein markers. The bands on the membranes represent the target of (**A**) CD276, (**B**) MCT4, (**C**) CD147, (**D**) EGFR and (**E**) CD133. Blue boxes indicate the bands that were cropped for representative images shown in the main text. The red box indicates the band that serves as the loading control, as mentioned in the main text.

**Supplementary Figure S9. Uncropped immunoblot images corresponding to Figure 2d.** Chemiluminescent images are merged with brightfield images of membranes to aid in the visualization of protein markers. The bands on the membranes represent the target of (**A**) CD276, (**B**) MCT4, (**C**) CD147, (**D**) EGFR and (**E**) CD133. Blue boxes indicate the bands that were cropped for representative images shown in the main text. The red box indicates the band that serves as the loading control, as mentioned in the main text.

**Supplementary Figure S10. Uncropped immunoblot images corresponding to Figure 3a and b.** Chemiluminescent images are merged with brightfield images of membranes to aid in the visualization of protein markers. The bands on the membranes represent (**A**) Figure 3a, (**B**) Figure 3b. Blue boxes indicate the bands that were cropped for representative images shown in the main text. The red box indicates the band that serves as the loading control, as mentioned in the main text. Blots underwent trimming before hybridization with antibodies.

**Supplementary Figure S11. Uncropped immunoblot images corresponding to Figure 4c and d.** Chemiluminescent images are merged with brightfield images of membranes to aid in the visualization of protein markers. Blue boxes indicate the bands that were cropped for representative images shown in the main text. The red box indicates the band that serves as the loading control, as mentioned in the main text.
